# Improvement of Periodontal Parameters with the Sole Use of Free Gingival Grafts in Orthodontic Patients: Correlation with Periodontal Indices. A 15-Month Clinical Study

**DOI:** 10.3390/ijerph17186578

**Published:** 2020-09-09

**Authors:** Marco Orsini, Dunia Benlloch, Juan José Aranda Macera, Karina Flores, José-Vicente Ríos-Santos, Francisco Javier Pedruelo, Blanca Ríos-Carrasco, Massimo di Cesare

**Affiliations:** 1 Private Practice Via Baldassarre Nardis, 67100 L’Aquila, Italy; duniabenlloch@gmail.com; 2 Department of Periodontology, School of Dentistry, Universidad Complutense, 28040 Madrid, Spain; juanjo.aranda@yahoo.es; 3 Department of Periodontology, School of Dentistry, Universidad de Sevilla, 41009 Seville, Spain; brios@us.es; 4 Private Practice Plaza Reyes Magos 10, 28007 Madrid, Spain; karinaifh@hotmail.com; 5 Private Practice Calle Pasiòn, 13, 47001 Valladolid, Spain; cipjpedruelo@hotmail.com; 6 Private Practice Via Patini, 67039 Sulmona, Italy; massimo.dicesare@gmail.com

**Keywords:** gingival grafts, orthodontics, periodontal index, free gingival grafts

## Abstract

The aim of this study was to evaluate the changes in periodontal parameters solely using free gingival grafts during orthodontic treatment without any oral hygiene re-enforcement. Methods: A total of 19 patients underwent periodontal examination before orthodontic treatment. Patients received oral hygiene instruction and professional hygiene therapy. Where needed; full periodontal treatment was completed. Only periodontally stable patients were included in the study. Periodontal indices and keratinized tissue were recorded at time 0 (T0) (delivery of orthodontic appliances), and at three months (T1) during orthodontic therapy; when surgery was performed. At T1; orthodontically treated sites with minimum keratinized tissue (≤1 mm) received a free gingival graft to enhance the band of keratinized tissue. At three months after surgery (T2), new measurements were recorded. The orthodontics-treated sites after three months (T1) were used as control. The same sites were used as a test three months after mucogingival correction (T2). Between T1 and T2; orthodontics was suspended; no professional oral hygiene was performed; and no additional oral hygiene instructions were given to the patient. No oral hygiene procedures were administered for 15 months (T3), when the final recordings were taken. Results: The results showed that there was a worsening of gingival index (GI) and plaque index (PI) of the treated sites between T0 and T1 during initial orthodontics treatment; whereas there was an improvement of the gingival inflammation at T2 when compared with T1. At T2; there was also a statistically significant improvement in GI and PI compared with T0. A T3 improvement in periodontal parameters was sustained. A non-parametric test (Wilcoxon signed-rank test) was used for statistical analysis. Conclusions: Augmentation of the width of keratinized gingiva; as the sole treatment; favors the improvement of GI and PI during orthodontic therapy.

## 1. Introduction

Some controversy still exists about the need for an adequate width of keratinized gingiva. There is a clinical impression that the presence of a certain width of keratinized tissue is important for maintenance of periodontal health and prevention of soft tissue recession. Lang and Löe suggested a minimum apico-coronal width of 2 mm of keratinized gingiva to maintain gingival health [1], corresponding to 1 mm of the attached portion of gingiva. However, several clinical and experimental investigations have demonstrated how the absence of attached keratinized tissue is compatible with the maintenance of periodontal health. There is no deterioration of gingival health and progression of gingival recession with a minimal band of gingiva, provided that traumatic tooth brushing and inflammation are controlled [2,3,4,5]. Furthermore, the presence of keratinized mucosa has not been demonstrated to be a crucial factor for the prognosis of fixed orthodontic therapy [6,7]. Thus, the presence of a narrow band of attached keratinized gingiva is not an indication for gingival augmentation by itself.

However, there are many clinical situations where mucogingival treatment may be considered. Orthodontic treatment may cause the recession of marginal tissue. Some studies have shown that a narrow band of attached gingiva is capable of withstanding orthodontic movement [8]. However, if teeth are moved outside the alveolar envelope, an alveolar bone dehiscence will result and, in the presence of a thin marginal tissue lined by buccal mucosa, there may be a higher risk of marginal tissue recession [7,8]. A thin gingiva might be less resistant in the presence of inflammation or tooth-brushing trauma.

Gingival augmentation can also be considered in situations where the absence of attached keratinized gingiva might compromise plaque control, thus leading to gingival inflammation. A movable gingival margin could possibly facilitate the introduction of plaque into the gingival crevice, making it difficult to detect and difficult to remove by conventional toothbrushing [1]. Thus, gingival augmentation may be considered in the presence of a high frenulum attachment, when vestibular depth is needed for treatment with removable partial dentures [9,10], and in all clinical situations where a change in the morphology of the mucogingival complex may contribute to the establishment of proper plaque control [1].

For patients who undergo fixed orthodontic treatment, the presence of thick attached keratinized tissue may constitute a protective factor against marginal tissue inflammation [7,8]. For the placement of a restoration in a subgingival site with a narrow gingiva, poor plaque control and severe gingival inflammation have been suggested to lead to gingival recession, although this has not been demonstrated in humans. The same situation can be found in patients with fixed orthodontic appliances, partially inside a sub-gingival site with a narrow gingiva, poor plaque control, and severe gingival inflammation [11,12,13,14].

Many techniques have been used to augment the dimensions of gingival tissue. Longitudinal studies have revealed that procedures using pedicle and free grafts are effective for this purpose [15,16,17]. Karring et al. performed a series of heterotopic gingival and alveolar mucosal transplants in an animal model. They found that the transplanted tissues always retained their original structure and specificity, even after one year [18]. The predictability of epithelialized free gingival autografts has been demonstrated through the proven stability of the newly created keratinized tissue for up to four years [2,3,15,16,18,19,20]. However, this grafting technique generally results in compromised esthetics (namely, a patch-like area).

In addition, free connective tissue grafts may be used due to a less invasive palatal wound and improved esthetic result. These seem to be predictable in gingival augmentation procedures [21]. However, follow-up data of up to six months have shown an important degree of shrinkage [22].

The aim of this study was to evaluate whether the sole augmentation of keratinized tissue is able to produce a variation of periodontal indices during orthodontics therapy without any additional issues. The following parameters were studied: (1) dimensional changes of free gingival grafts and (2) inflammatory status of the marginal gingival before and after mucogingival correction through the recording of gingival index (GI), plaque index (PI), probing pocket depth (PPD), and clinical attachment Level (CAL).

## 2. Materials and Methods

A total of 19 patients were used for the present study. The patients were 8 men and 11 women, between 30 and 52 years of age. None of them were smokers (Table 1).

All patients underwent a full-mouth dental and periodontal examination. All patients filled in a routine questionnaire from which the following data were obtained: general medical history, age, sex, and smoking habits.

All patients were systemically healthy and without any significant history of systemic diseases. Patients with any condition that could affect gingival status or healing were excluded. All patients were informed and provided their written consent for the procedures to be performed (PEIBA authorization—Ethics Portal of Biomedical Research in Andalusia), code 1259–N–19. Before initiation of orthodontic treatment, all patients underwent full periodontal examination. All periodontal parameters were collected at the site of minimum amount of keratinized tissue using a North Carolina University probe (CP–15UNC—Hu-Friedy, Chicago, IL, USA). Standardization and reproducibility of the recordings were accomplished by the use of a customized stent [23]. The probe was pre-marked at the first examination. Thus, the same probe was always used in the same patient for all the measurements at all time intervals (T0, T1, T2, and T3). The same examiner (fully-trained periodontist) recorded all the measurements (DB). All patients who underwent surgical treatment had an amount of keratinized tissue ≤ 1 mm. Gingival index (GI, a method for assessing the severity and the quantity of gingival inflammation with a score scale from 0 to 3) [24]; Plaque Index (PI, the quantity of plaque on a tooth with a score scale from 0 to 3) [25]; probing depth (PPD, the distance from the gingival margin to the bottom of the probable crevice (i.e., where the probe tip stops) [26,27]; and width of keratinized gingiva were recorded, respectively. All patients received oral hygiene instruction (toothbrush, a rubber tip and, when needed, an interproximal brush was given), and professional hygiene therapy. Where needed, full periodontal treatment was provided.

Orthodontic treatment was started three months after completion of periodontal therapy and re-evaluation of periodontal status (T0). Only patients who were periodontally stable were included in the study. Fixed vestibular no-nickel orthodontic appliances were clinically acceptable regarding materials, finishing of composite, and bracket, and everything was placed out of the gingiva. Single band and fixed orthodontic appliance were included. The Roth technique was used, slot 022 (bkt Equilibrium Dentaurum Gmbh and Co., Ispringen, Germany). The sequence of wires was 0.14, 0.16, 0.16 × 0.22 (Nitinol Dentaurum Gmbh and Co., Ispringen, Germany). The orthodontic treatment was always performed by the same practitioner (MDC).

At the time of starting orthodontic treatment at T0, all patients received new oral hygiene instructions.

Periodontal indices were recorded at T0, at delivery of orthodontic appliances, and again after three months (T1) during orthodontic therapy and before surgery. At T1, orthodontically treated sites with minimum amount of keratinized tissue (≤1 mm) received a free gingival graft to enhance the band of keratinized and attached tissue. At three months after surgical correction (T2), a final assessment of the treated sites was recorded. The orthodontic treatment was continued until its completion after T2. At all interval times, full mouth scores were recorded [28].

The orthodontically treated sites after three months (T1) were used as control. The same sites were used as test sites three months after mucogingival correction (T2). Between T1 and T2, active orthodontics therapy was suspended without removal of the appliance and no professional oral hygiene was performed, nor were additional oral hygiene instructions given to the patient. The orthodontics treatment was continued until its completion after T2. No professional hygiene was carried out between T2 and T3. At 15 months (T3), new recordings were taken.

At T0, no periodontal pockets or bleeding on probing were present. The full mouth plaque (FMS) score was ~10%. FMS was recorded at T1, T2, and T3. The width of keratinized gingiva was measured from the free gingival margin to the mucogingival junction with a North Carolina University probe.

Free gingival autografts were used to augment keratinized tissue in the selected sites. The size of the free gingival grafts was recorded. Photographs (D5500 Medical Nikkor 120mm Nikon Corporation, Tokyo, Japan) were taken at beginning of orthodontics therapy (T0), at surgical time (T1), and 3 (T2) and 15 months (T3) after surgery.

All surgeries were performed by the same operator (MO). The surgical technique employed in the test group consisted of the following steps:

- Using a #15 blade, an intrasulcular incision was made and a partial thickness flap was raised. The recipient site was prepared by sharp dissection to create a bleeding periosteal bed free of all muscle attachments;
- The resulting flap was excised or sutured at the base of the newly created vestibule with 5-0 plain gut T-mattress sutures (Ethicon, LLC—Johnson and Johnson Co., Guaynabo, Puerto Rico).

An area in the palate, just distal to the rugae palatinae and about 2 mm away from the gingival margin, was chosen as the donor site. The size of the graft to be harvested was predetermined using a tin foil stent prepared over the recipient site. With sharp dissection, the graft tissue was harvested. Care was taken to remove a homogeneous thickness graft of about 1.5–2 mm thick. The palate was sutured back with 4–0 silk (Ethicon, LLC—Johnson and Johnson Co., Guaynabo, Puerto Rico) to provide compression and minimize post-operative bleeding. Pressure was applied for about five minutes.

1. The graft was measured with the probe and its measurements recorded;
2. The graft was sutured over the recipient bed with 5-0 absorbable interrupted single sutures (Plain Gut Ethicon, LLC—Johnson and Johnson Co., Guaynabo, Puerto Rico). Where necessary, one or more cross-horizontal mattress sutures were used to ensure the complete stabilization of the graft. The area was then covered with oxidized regenerated cellulose (Tabotamp Ethicon, LLC, city, Johnson and Johnson Co., Guaynabo, Puerto Rico), adhesive dry foil (Burlew Dryfoil Jelenko, Armonk, NY, USA) and periodontal dressing (Coe Pak GC America Inc., Alsip, IL, USA);
3. Post-operative instructions were given to all patients. Chlorhexidine 0.12% rinses were prescribed twice a day for two weeks. Anti-inflammatory therapy was prescribed for pain control (Ibuprofen 400 mg b.i.d. for one week);
4. Palatal sutures and periodontal dressing were removed at one week; photographs and graft measurements were recorded.

At three months after surgery, PI, GI, PPD, CAL, and width of keratinized tissue were recorded, and new photographs were taken. Between T1 and T2, oral hygiene procedures were not reinforced, and no maintenance treatment was performed throughout the period of study.

All measurements taken at three months were repeated at 15 months.

**Figure 1:**
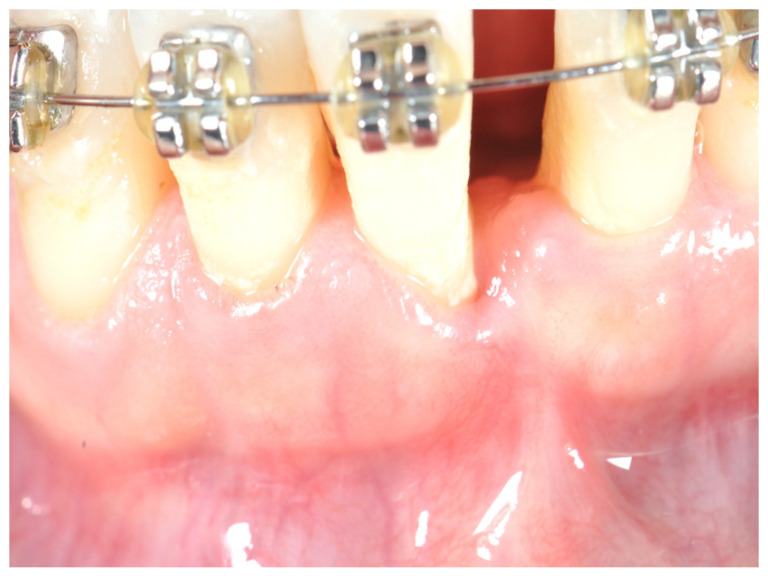
Initial situation at T0: Patient with lack of keratinized tissue and frenum pull on tooth #25.

**Figure 2:**
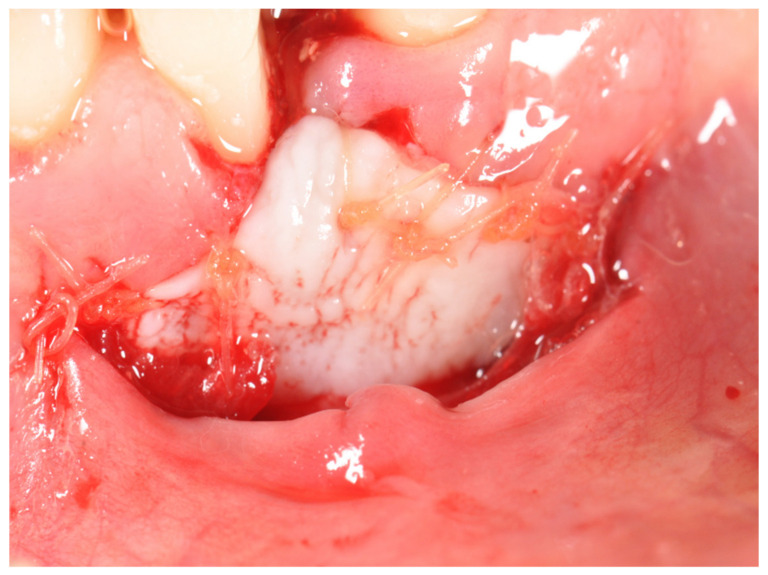
Free gingival graft is adapted and sutured on the recipient site with absorbable sutures.

**Figure 3:**
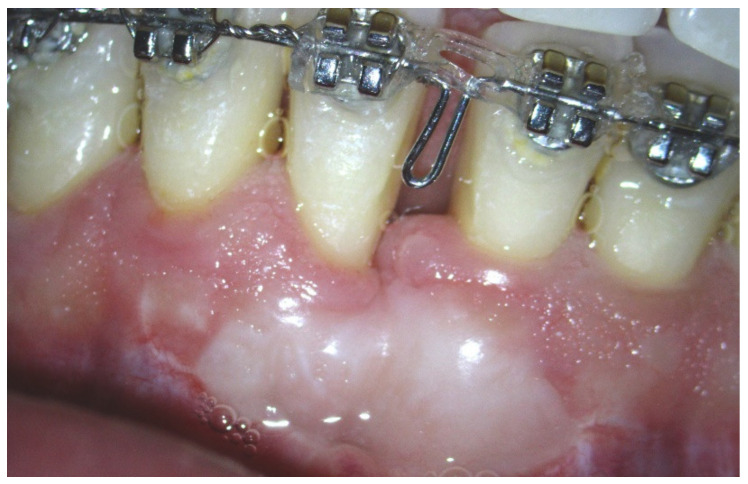
Clinical situation at T2 before starting completion of orthodontic treatment.

**Figure 4:**
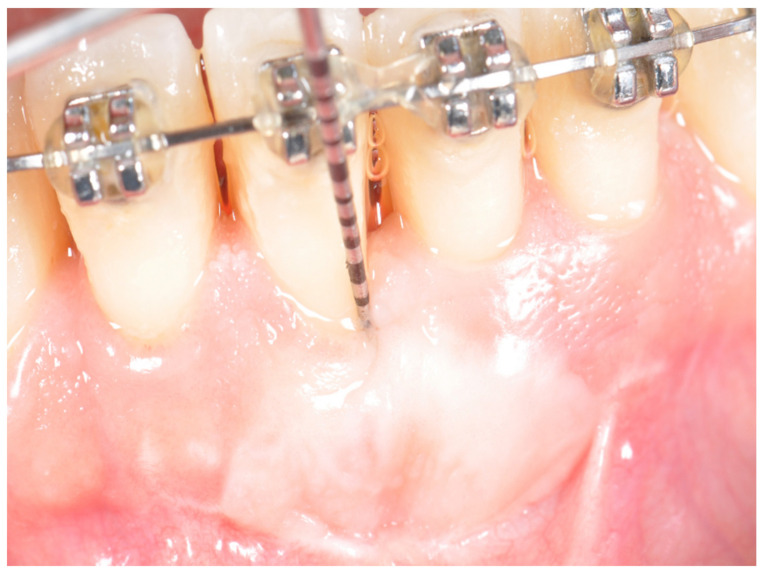
Final haling at completion of orthodontics therapy (T3). Frenum has been eliminated and the band of keratinized attached gingival augmented.

When present, the pre-existing keratinized gingiva was completely eliminated during the surgical procedure and the amount of keratinized tissue at baseline was always recorded as 0 mm. We then considered that any keratinized tissue present at the following appointments was the result of the free gingival graft keratinization. So, measurements of the size of the graft and measurements of the width of keratinized tissue were considered equivalent in this group of patients. We used the term size of the graft [22] for the observations at T1. The width of keratinized tissue [22] was used for the observations at T2 and T3, as keratinized tissue was already present. The dimensional changes in size of the graft/width of keratinized tissue were termed as shrinkage of the graft [22] (Figure 1, Figure 2, Figure 3 and Figure 4).

A non-parametric test (Wilcoxon signed-rank test) was used to assess and compare the changes of GI, PI, PPD, and CAL between T0 and T1, T1 and T2, T0 and T2, T0 and T3, and T1 and T3. Shrinkage of the graft between T1 and T2 and T1 and T3 was also assessed.

The sample size computation was done as follows: the main comparison of the study was the difference in GI between T1 and T2. Based upon the assumption of a mean T1 GI = 1.10, a mean T2 GI = 0.50, a standard deviation of the mean difference = 0.80, a two-sided alpha = 0.05, 16 subjects were required to achieve 80% statistical power. Conservatively assuming 15% missing data or lost to follow-up, we enrolled a total of 19 subjects.

## 3. Results

The grafted area and the donor site healed uneventfully in all cases. The recordings of GI, PI, PPD, CAL, and width of keratinized tissue at T0, T1, T2, and T3 are provided in Table 2 and Table 3. The mean GI at T0 was 1.05 ± 0.62, at the time of surgery T1: 1.58 ± 0.51, at T2 3 months after surgery: 0.37 ± 0.5, and at T3 (15 months) 0.63 ± 0.5 (Table 2). The mean PI at T0 was 1.16 ± 0.37, at the time of surgery T1: 1.79 ± 0.54, at T2: 0.84 ± 0.37, and at T3 0.95 ± 0.40 (Table 2). Both GI and PI showed a statistically significant worsening between T1 and T0 (*p* = 0.007 for GI; *p* = 0.002 for PI), but a statistically significant improvement between T1 and T2 (*p* < 0.001 for GI and *p* < 0.001 for PI). In addition, we detected a statistically significant improvement in GI (*p* = 0.006) and PI (*p* = 0.03) between T2 and T0 (*p* = 0.03) (Table 2). At T3 versus T1, improvements in GI *(p* < 0.001) and PI *(p* = < 0.001) were sustained.

Seventeen patients showed an improvement in GI from T1 to T2 and 16 patients showed an improvement in PI during the same period. In none of the patients could a worsening of the inflammatory status be detected after mucogingival correction at T2 or at T3. Thus, we considered that inflammation was less evident when gingival augmentation was performed and that plaque control improved.

No statistically significant differences at any time of the study could be detected for PPD or CAL (Table 3).

The full mouth plaque scores [22] at T0, T1, T2, and T3 were 10%, 13%, 12%, and 13%, respectively.

The statistical analysis also demonstrated a significant shrinkage at three and 15 months (T2 and T3, respectively) of the graft compared to baseline. When we analyzed the dimensional changes of the graft in detail, we observed that shrinkage of the graft was 30.8% ± 8.04%. The mean size of the graft was 9.25 ± 2.09 mm at the time of surgery (T1) with a contraction of 2.83 ± 1.03 mm after three months (T2) and 3.32 ± 1.20 at 15 months (T3) (Table 3).

## 4. Discussion

The main purpose of the present study was to evaluate any changes in the keratinized tissue during orthodontic therapy. The data on shrinkage of the graft were presented for completion of the description of the investigation.

Here, the same site in the same patient was used both as control and test. All periodontal treatments were accomplished three months before starting the orthodontic therapy and six months before surgical procedure. In addition, oral hygiene instructions were given three months before T0 and six months before T1. No additional periodontal therapy or oral hygiene re-enforcement was performed during T0 and T2. As such, we were able to independently evaluate the changes in periodontal parameters due to the only variable determined by the augmentation of the attached keratinized tissue. This also allowed us to eliminate any intra- and inter-individual variations. The choice of a different site as control in the same or in a different patient would not have given us the opportunity to control all the intra-individual variables in each subject throughout the investigation. Standardization of the periodontally related inflammatory parameters was accomplished at the beginning of the study with all patients having a full mouth plaque score [28] of ≤10%.

Gingival keratinized tissue is usually augmented by free gingival graft [10,15,16,17,18,19,20,21,22,23,24,25,26,27,28,29] or free connective tissue grafts. Edel demonstrated that the use of free connective tissue grafts might be a feasible alternative procedure [21]. In the present study, a free epithelialized gingival graft was used to minimize shrinkage and favor higher predictability of the final dimension of the keratinized attached tissue [16,17]. Connective tissue grafts have a higher degree of contraction compared with epithelialized tissue [22]. It is clinically evident that epithelialized free gingival grafts create a patch-like area at the recipient site, without blending totally into the adjacent tissue. As already postulated, the genetic information for keratinization resides in the lamina propria of the connective tissue. It seems that gingival color information might reside in the external epithelial cells. However, all the sites treated in the present investigation were in the mandible, where aesthetics is less of a concern for the patients. This resulted a total mean shrinkage of the graft of 30.8% three months after surgery. This value is in concordance with the shrinkage reported for epithelialized free grafts in gingival augmentation procedures (25% at four years follow-up [14] even though the measurements were taken only at three months in the present investigation. In a previous study by Orsini et al., shrinkage was reported to be more evident between surgery and at four weeks. Shrinkage occurs during the first year and then the width of keratinized tissue remains stable after that period of time [17,22]. As such, some additional shrinkage can be expected in the follow up.

As for the periodontal indices, the following observations can be made. GI and PI showed a statistically significant improvement after the augmentation of the band of keratinized tissue during the presence of an orthodontic fixed appliance. A better inflammatory status of the gingiva of the grafted area was detected, regardless any oral hygiene procedure or re-enforcement of oral hygiene instructions. The changes in GI and PI detected in the present study showed a worsening of the inflammatory status of sites with an absence or a ≤1 mm band of keratinized tissue accompanied or not by the lack of vestibular depth when an orthodontic fixed appliance was delivered.

The fixed appliance might have caused lower efficiency in the daily patients’ oral hygiene maneuvers. When looking at the whole mouth, where the quality and quantity of gingival tissue did not change, no significant changes were detected in the full plaque score at any of the time intervals. There was a very light increase between T0 and T1, which remained stable at T2 and T3.

When present, the frenum was removed to permit preparation of the recipient bed and increase the vestibular depth of the attached keratinized tissue. However, mucogingival correction with frenectomy is not justified to improve plaque control and gingivitis in the presence of vestibular depth and an adequate band of keratinized tissue [29,30,31,32,33].

Alterations in gingival dimensions are correlated in the direction of tooth movement. Vestibular movements can result in a thinning of periodontal support, while lingual movements show an increase of gingival dimensions [8].

Some investigations showed that it is important to consider the need for facial dental movement in a zone with a minimal thickness of bone and thin gingival tissue. Clinical situations like this may indicate pre-orthodontic treatment to augment keratinized attached tissue [8,11,34]. It has been suggested that the orthodontic repositioning of a tooth out of the alveolar bone housing with a vestibular movement producing a radicular bone dehiscence may constitute a risk factor for marginal tissue apical migration, especially in sites with thin gingiva [33]. The presence of inflammation at these thin gingiva sites may worsen the process of gingival tissue recession [35,36,37].

In the present study, sites with ≤1 mm keratinized tissue showed significative improvements in the inflammatory parameters when treated with a graft to increase the band of keratinized gingiva with the presence of an orthodontia appliance. Thus, even if the aim of the present study was to evaluate whether we could modify some gingival inflammatory parameters by the sole use of free gingival grafts in the presence of orthodontia appliances, we suggest that the increase in gingiva in sites with <1 mm of keratinized tissue may contribute to minimizing the risk of marginal apical gingival migration when performed before facial dental movement outside the alveolar bone housing. A limitation of the present study was the lack of data to correlate possible changes which were due to dental movement lingually or facially between T2 and T3, besides the presence of keratinized tissue. We speculate that improvement of dental position at this time interval also contributed to the substantial results accomplished in the T1–T2 interval.

At T3 compared with T0, CAL almost showed a statistical significance, although not completely (*p* = 0.054). We do not have enough data to relate this to root coverage or to the final dental position after therapy.

A careful control of oral hygiene parameters is important, especially for orthodontic patients, as both vestibular [38] and lingual [39] appliances are shown to alter the oral environment during treatment.

## 5. Conclusions

From these data, we speculate that in patients with fixed orthodontic appliances and sites with lack of keratinized tissue and vestibular depth, increasing the band of keratinized tissue and deepening of vestibular depth might favor plaque control and a minor degree of gingival inflammation.

Within the limitations of this study, this would justify the use of gingival augmentation procedures in orthodontic patients in sites with insufficient keratinized gingiva and/or shallow or absent vestibule, inadequate plaque control and/or progressive recession.

Further studies are needed to confirm the findings of the present investigation.

## Figures and Tables

**Table 1:** Sample data.

Patients	
Age (Years), Mean ± SD (Range)	41 ± 6.8 (30–52)
Sex (Female/Male)	11/9
Smokers	None
Sites	
Anterior/Posterior	19/0
Maxilla/Mandible	0/19

**Table 2:** Mean (SD) of gingival index (GI) and plaque index (PI) at T0, T1, and T2.

No.	Gingival Index				Plaque Index			
	T0	T1	T2	T3	T0	T1	T2	T3
1	1	2	1	1	1	2	1	1
2	1	1	1	1	1	2	1	1
3	0	2	1	1	1	2	1	1
4	1	2	0	0	1	3	1	1
5	1	1	0	0	1	1	1	1
6	1	2	0	1	1	2	1	1
7	1	1	0	0	1	1	1	1
8	1	2	1	1	1	2	1	1
9	1	2	0	1	1	2	1	1
10	2	2	0	0	2	2	1	1
11	1	1	0	1	1	2	0	0
12	0	1	0	0	1	1	1	1
13	2	2	1	1	2	2	1	1
14	1	1	0	0	1	1	0	1
15	1	2	0	1	1	2	1	2
16	0	1	1	1	1	2	1	1
17	2	2	0	1	2	1	0	0
18	1	2	1	1	1	2	1	1
19	2	1	0	0	1	2	1	1
MEAN	1.05	1.58	0.37	0.63	1.16	1.79	0.84	0.95
SD	0.62	0.51	0.50	0.50	0.37	0.54	0.37	0.40
Mean Differences (SD)								
	Gingival Index				Plaque Index			
	T1–T0	T2–T0	T2–T1	T3–T1	T1–T0	T2–T0	T2–T1	T3–T1
Mean	−0.53	0.68	1.21	0.95	−0.63	0.32	0.95	0.84
SD	0.70	0.89	0.63	0.52	0.68	0.58	0.52	0.60
P *	0.007	0.006	<0.001	<0.001	0.002	0.03	<0.001	<0.001
% of Subjects with 1 Unit Score Decrease								
	T1–T0	T2–T0	T2–T1		T1–T0	T2–T0	T2–T1	
	O.O	58.3	91.7		0.0	16.7	75.0	

Mean (SD) of Gingival Index and Plaque Index at T0, T1 and T2. * Wilcoxon matched-pairs test.

**Table 3:** Probing pocket depth (PPD), clinical attachment level (CAL), and graft width evolution.

	PPD	P *	CAL	P *	Graft Width	P *
Mean (SD)						
T0–Baseline	2.26 (0.65)		2.58 (1.02)			
T1–Preintervention	2.52 (0.51)		2.74 (0.99)		9.25 (2.09)	
T2–3 Months Postintervention	2.75 (0.87)		2.83 (1.40)		6.42 (1.62)	
T3–15 Months Postintervention	2.63 (0.76)		3.00 (1.15)			
Mean Differences (SD)						
T1–T0	0.33 (0.78)	0.2	0.16 (0.83)	0.3		
T2–T0	0.42 (0.79)	0.1	0.25 (0.62)	0.2		
T2–T1	0.08 (0.67)	0.7	0.17 (0.58)	0.3	−2.83 (1.03)	0.002
T3–T0	0.37 (0.76)	0.054	0.42 (0.69)	0.019		
T3–T1	0.11 (0.66)	0.5	0.26 (0.65)	0.1	−3.32 (1.20)	<0.001

* Wilcoxon matched-pairs test.
